# Intranasal Immunization with Pressure Inactivated Avian Influenza Elicits Cellular and Humoral Responses in Mice

**DOI:** 10.1371/journal.pone.0128785

**Published:** 2015-06-09

**Authors:** Shana P. C. Barroso, Dirlei Nico, Danielle Nascimento, Ana Clara V. Santos, José Nelson S. S. Couceiro, Fernando A. Bozza, Ana M. A. Ferreira, Davis F. Ferreira, Clarisa B. Palatnik-de-Sousa, Thiago Moreno L. Souza, Andre M. O. Gomes, Jerson L. Silva, Andréa C. Oliveira

**Affiliations:** 1 Instituto de Bioquímica Médica Leopoldo de Meis, Universidade Federal do Rio de Janeiro, Rio de Janeiro, Rio de Janeiro, 21941–902, Brazil; 2 Instituto Nacional de Ciência e Tecnologia de Biologia Estrutural e Bioimagem, Rio de Janeiro, Brazil; 3 Instituto de Microbiologia Paulo Góes, Universidade Federal do Rio de Janeiro, Rio de Janeiro, Rio de Janeiro 21941–590, Brazil; 4 Fundação de Pesquisa Clínica Evandro Chagas, Fundação Oswaldo Cruz (FIOCRUZ), Rio de Janeiro, Rio de Janeiro, Brazil; 5 Laboratório de Vírus Respiratórios, WHO/NIC, Instituto Oswaldo Cruz/FIOCRUZ, Rio de Janeiro, Rio de Janeiro, Brazil; Thomas Jefferson University, UNITED STATES

## Abstract

Influenza viruses pose a serious global health threat, particularly in light of newly emerging strains, such as the avian influenza H5N1 and H7N9 viruses. Vaccination remains the primary method for preventing acquiring influenza or for avoiding developing serious complications related to the disease. Vaccinations based on inactivated split virus vaccines or on chemically inactivated whole virus have some important drawbacks, including changes in the immunogenic properties of the virus. To induce a greater mucosal immune response, intranasally administered vaccines are highly desired as they not only prevent disease but can also block the infection at its primary site. To avoid these drawbacks, hydrostatic pressure has been used as a potential method for viral inactivation and vaccine production. In this study, we show that hydrostatic pressure inactivates the avian influenza A H3N8 virus, while still maintaining hemagglutinin and neuraminidase functionalities. Challenged vaccinated animals showed no disease signs (ruffled fur, lethargy, weight loss, and huddling). Similarly, these animals showed less Evans Blue dye leakage and lower cell counts in their bronchoalveolar lavage fluid compared with the challenged non-vaccinated group. We found that the whole inactivated particles were capable of generating a neutralizing antibody response in serum, and IgA was also found in nasal mucosa and feces. After the vaccination and challenge we observed Th1/Th2 cytokine secretion with a prevalence of IFN-γ. Our data indicate that the animals present a satisfactory immune response after vaccination and are protected against infection. Our results may pave the way for the development of a novel pressure-based vaccine against influenza virus.

## Introduction

Influenza viruses pose a serious global health threat, particularly in light of the newly emerging strains, such as the avian H5N1 and H7N9 viruses [1–3]. Influenza virus causes respiratory infections resulting in great human and animal suffering and substantial economic losses [4]. In humans, it is responsible for 3–5 million clinical infections and 250,000–500,000 deaths annually worldwide [5,6]. In recent years, there has been a sharp increase in the number of outbreaks of avian influenza in poultry, which has been associated with several avian influenza outbreaks in humans [7,8].

Vaccination remains the primary method to prevent acquiring influenza or to avoid developing serious complications related to the disease. Vaccination is the most cost-effective method for preventing economic losses and for decreasing influenza-related morbidity and mortality in humans and animals [9].

Virus-inactivated split vaccines induce the production of antibodies against the globular head of the hemagglutinin [10,11]. Most current influenza vaccines are split vaccines aimed at inducing a neutralizing antibody immune response. These vaccines are specific according to the subtype and often the strain, thus the vaccines based on this strategy require accurate prediction of the circulating viral strains during an influenza outbreak. Unfortunately, such accurate predictions are not feasible [12]. Most current avian vaccines, which are primarily based on the chemically inactivated whole virus [13], have some important drawbacks, such as the risk of partial inactivation of the virus, a change in the immunogenic properties of the virus, and the toxicity of the inactivating agent [14,15]. Hydrostatic pressure (HP) inactivation does not introduce exogenous substances into the vaccine and usually results in highly immunogenic preparations. In addition, this process is straightforward when considering large-scale immunization [16,17].

An ideal vaccine would stimulate the production of CD8^+^ T and CD4^+^ T cells, a cytokine response, IgA production in the nasal mucosa, a longer lasting immune response, and cross protection. Individuals vaccinated parenterally with an inactivated virus develop a rapid systemic immune response in the blood and a weak mucosal immune response. To induce a greater mucosal immune response, vaccines introduced by intranasal administration are highly desired because vaccination via this route can not only prevent disease but can also block an infection at its primary site [18].

Hydrostatic pressure (HP) is a non-thermal, energy-efficient technology that has been used as a potential method for viral inactivation and feasible vaccine production. It allows for the control of the dissociation of oligomeric proteins of virus particles [19–21]. HP has been shown to cause structural changes in some viruses [22], preserving covalent bonds [23] while interfering with their infectivity [24] and triggering humoral immunological responses in their recipients [25].

Some studies discuss the use of avian subtypes for human vaccination [26,27], since the avian and human influenza virus homologues possess epitopes that are located on the internal viral proteins and nucleoproteins. Thus, cellular immunity induced by infection with an avian virus could confer heterosubtipic protection against human influenza viruses [28].

Our study model, the avian influenza A H3N8 subtype, has infection spectrum to birds, horses, dogs and camels. A pressing concern is the infection of pet dogs, a primary companion for humans. This raises the possibility that dogs may provide a new source for transmission of novel influenza A viruses to humans [29, 30].

Here, we investigated the effect of HP on inactivating the H3N8 virus. We also asked whether the pressure-inactivated virus would be able to promote humoral and cellular responses. Lastly, we asked whether the pressure-inactivated virus was capable of protecting challenged animals. We found that HP completely abolishes H3N8 virus infectivity while still maintaining hemagglutination and neuraminidase functionalities. Our results may pave the way for the development of a novel pressure-based vaccine against influenza viruses.

## Material and Methods

### Ethics statements

All mouse studies followed the guidelines set by the National Institutes of Health, USA. The animals used in the studies received humane care in compliance with the *Principles of Laboratory Animal Care* formulated by the National Society for Medical Research and the *Guide for the Care and Use of Laboratory Animals* prepared by the US National Academy of Sciences.

The Institucional Animal Care Committee (Comissão de Ética com Uso de Animais (CEUA) em Experimentação Científica do Centro de Ciências da Saúde da Universidade Federal do Rio de Janeiro) approved the animal protocols (UFRJ, Brazil, protocol IBQM059).

Written informed consent from the donor was obtained for use of the erythrocyte samples in the hemagglutination assays. These protocols received approval from the Ethics Committee of University Hospital from the Federal University of Rio de Janeiro, Brazil (Comitê de Ética em Pesquisa do Hospital Universitário Clementino Fraga Filho, CAAE number 03102012.4.1001.5257).

### Mice

Six-week-old female BALB/c mice, obtained from the Laboratory Animals Breeding Center, Oswaldo Cruz Foundation (CECAL/FIOCRUZ) were used in this study.

Animals were caged in microisolator cages with food and filtered fresh water *ad libitum* in a room at 24°C and a 12-h light-dark cycle. All manipulation of mice and cages was performed in the laminar flow hood (Class II Biological Safety Cabinet).

Animals were monitored for signs of disease daily (including changes in body weight). Each treatment group was used only once. All efforts were made to minimize suffering. All animal experiments were performed in the afternoon. Mice were euthanized if they lost 20% of their body weight after vaccination and/or challenge.

### Influenza virus preparation

The virus purification was performed as described by Rovozzo and Burke [31]. Briefly, an avian influenza A/duck/Ukraine/1/63 (H3N8) virus sample was replicated for 24 h at 37°C in the allantoic cavity of 10-day-old embryonated chicken eggs. The allantoic fluid was collected, and the cell debris was removed by a low speed spin (6,000 × *g*). The virus was pelleted by spinning the allantoic fluid at 80,000 × *g* and was then resuspended in TE buffer (20 mM Tris, 2 mM EDTA, pH 8.4) and banded at 100,000 × *g* on a continuous 20–60% sucrose density gradient in TE buffer, pH 8.4. The protein concentration of the virus samples was determined as described by Lowry *et al*. [32], and the samples were then stored at -80°C.

### Assay for the detection of the residual virus infectivity in cell culture and embryonated eggs

The residual infectivity of the pressurized virus samples was assayed for three sequential serial passages in embryonated chicken eggs and monolayers of Madin-Darby canine kidney (MDCK) cells. For each blind passage, the samples showed no infectivity by the hemagglutination assay or the TCID_50_. Each assay was performed three times to assure the reproducibility of the results.

### Cell culture and the TCID_50_

MDCK cells were cultured in low glucose Dulbecco's modified Eagle medium (DMEM; Invitrogen) supplemented with 10% fetal bovine serum (Invitrogen, Carlsbad, CA, USA). Before the infection, 80–90% confluent cells were washed with PBS to remove the FBS and were infected with 100 μg of the virus diluted in serum-free DMEM with 2 μg of trypsin and incubated for 1 h at 37°C. The infection medium was removed, and the cells were washed with PBS. The period of infection was 48 h. The infectivity of the influenza virus was studied by a determination of the 50% tissue culture infectious dose (TCID_50_) in the MDCK cells. The cells were infected with serial dilutions of 10^-1^ to 10^-8^. After 48 h at 37°C, the cytopathic effects of influenza virus were observed under a microscope, and the TCID_50_ was calculated according to the Reed and Muench method [33].

### Hemagglutination assay

Virus preparations were assayed for their hemagglutinating activity in 96-well micro-titer plates (U type, Nunc, Roskilde, Denmark). A total of 25 μL of the viral suspension was added to the first well in column one and serial dilutions were made. The positive control was performed with lectin, and the negative control was performed with PBS. Lastly, 25 μL of a 0.5%human erythrocyte suspension was added to each well of the plate and hemagglutinating titers were recorded after 45 min as described previously by Hierholzer and Killington [34].

### Sialidase assay

Virus preparations were assayed for sialidase activity in 96-well micro-titer plates (black flat-bottom type, Corning, NY, USA) with 4-methylumbelliferyl-N-acetyl-α-D-neuraminic acid (4-MU-NANA) ammonium salt (Nacalai Tesque, Kyoto, Japan) according to a fluorometric assay method as described previously by Song and colleagues [35]. Five microliters of the viral suspension, 20 μL of the NA inhibitor solution, and 20 μL of the 0.1 mM 4-MU-NANA solution were mixed and incubated for 60 min at 37°C. The fluorescence of the released 4-MU was measured with the excitation at 365 nm and emission spectra were recorded at 450 nm in a SpectraMax M5 fluorescence spectrophotometer (Molecular Devices, California, USA). Relative activities were calculated as described by Song and collaborators [35].

### Electron microscopy

The visualization of the pressurized and control viruses was performed using a Morgani transmission electron microscope operated at 100 kV. The samples (400 μg/mL) were placed in a 400 mesh copper grid coated with carbon, rinsed 3 times with water, and contrasted with 2% uranyl acetate.

### Vaccine preparation

An egg-grown inactivated H3N8 whole virus vaccine was prepared using hydrostatic pressure as the inactivating agent. Allantoic fluid was clarified and concentrated by ultracentrifugation. The viruses were inactivated by pressurization for 12 h at 282.7 MPa at 25°C.

### HP apparatus and procedure

The HP pump used in this study has a cylindrical body and is made of Vascomax 300. The samples were placed in the interior of the pump in a polyethylene tube with a volume of 1.5 mL. The pump is a manual pressure generator and was designed for applications in which a liquid is compressed within a small volume. It was purchased from ISS (Champaign, IL,USA). The experiment was started when a pressure of 282.7 MPa was reached. The pump was coupled to a thermostatic bath that maintained the temperature of the sample at 25°C. To monitor the cell temperature, a conduit for a thermometer was drilled into the pump. Ethanol was used as the pressure-transmitting fluid. Additional details concerning the HP equipment can be found in the report of Paladini and Weber [36].

### Prophylactic immunization, challenge, and the assessment of the immune response in mice

Six-week-old female BALB/c mice (10 per group) were immunized intranasally at weekly intervals, with 3 doses corresponding to 10^4.5^ TCID_50_ (3 μg per dose of the pressurized virus). On day 21, the mice were challenged via the intranasal route with 4 - 10^4.5^TCID_50_ of wild-type H3N8 virus.

One week post-vaccination and two weeks after challenge, the sera were collected. One and 12 weeks post-vaccination, feces and nasal washes were collected. These samples were used for the anti-influenza antibody assay. Additionally, cellular immunity was assessed by a cytokine ELISA assay of splenocyte supernatants, after the complete immunization and challenge. All of the experiments were performed before vaccination, one week after vaccination, and two weeks after challenge. Blood sampling were performed under 4% isoflurane in oxygen anesthesia. Euthanasia was performed under overdose of isoflurane in oxygen. Death was confirmed by personnel trained to recognize cessation of vital signs in euthanized mice. A combination of criteria was used to confirm death, including lack of pulse, breathing, corneal reflex, inability to hear respiratory sounds and heartbeat by use of a stethoscope, graying of the mucous membranes, and rigor mortis.

### Determination of virus titers in lungs

Groups of six BALB/c mice were vaccinated with pressurized virus or saline (as a control) and challenged by the intranasal route. On days 3 and 6 post-challenge, the animals`lungs were removed for quantification of virus. Briefly, lung tissue was homogenized in PBS containing 1% BSA. The homogenates were spun at 1000 × *g* to remove cellular debris, and the supernatants were used for virus quantification. Titers are expressed as TCID_50_ per milliliter of lung homogenate in MDCK cells. The results are expressed as the means of 6 animals.

### Viral quantification by qRT-PCR

When virus titration was not feasible due to low amounts of sample, influenza RNA levels were quantified by real-time RT-PCR. This was performed for the nasal washes of placebo and vaccinated animals. RNA was extracted with a commercial kit (RNeasy mini kit; Qiagen). All reagents for one-step real-time RT-PCR (qRT-PCR), including primers, probes and enzymes, were used as recommended by the World Health Organization (WHO) [37]. Virus quantification was based on a standard curve, as previously described [38]. In brief, RT-PCR with RNA from experimental assays was performed in parallel with serial 10-fold dilutions of PET26b+ plasmid (Novagen) containing an influenza M1 synthetic gene insert (Genescript) flanked by the plasmid`s XhoI and HindIII restriction sites. Quantification was expressed as copies/mL (http://cels.uri.edu/gsc/cndna.html). Negative controls without template were included in all reactions.

### Detection of influenza-specific antibodies

Antibodies were measured in sera, feces, and nasal washes using an ELISA. The ELISA assay used 2 μg of A/duck/Ukraine/1/63 (H3N8) per well and goat anti-mouse IgA, IgG1, and IgG2a horseradish peroxidase-conjugated antibodies (Southern, Biotechnology Associates, Birmingham, AL, USA) in a 1:1,000 dilution in blocking buffer. The reaction was developed with O-phenyldiamine (Sigma-Aldrich Co), stopped with 1 N sulfuric acid, and read at 492 nm. Each individual serum sample was analyzed in triplicate in double-blind tests. Positive and negative control sera were included in each test. The results are expressed as the mean of the absorbance values (492 nm) of the 1/100 diluted sera of each animal.

### Hemagglutination Inhibition (HI) assay

Neutralizing antibodies were also measured in sera using an HI assay, as described in the WHO Manual on Animal Influenza Diagnosis and Surveillance [39]. Briefly, the serum samples were serially diluted in PBS and were then mixed with aliquots of virus, corresponding to eight HA units, in V-bottom 96-well plates (Nunc, Roskilde, Denmark) and incubated for 60 min at room temperature. At the end of the incubation, 1.0% human red blood cells were added and incubated for a minimum of 30 min. The serum HI antibody titer of a given sample was defined as the reciprocal of the last serum dilution that completely inhibited the hemagglutination.

### Analysis of cytokines

All further analyses of the cellular immune response were carried out using 10^6^ splenocytes after 5 days of *in vitro* culturing at 37°C and 5% CO_2_ in supplemented RPMI medium and/or 10 μg of influenza virus (*in vitro* re-stimulation).

The secretion of IFN-γ, TNF-α, IL-2, IL-4, and IL-6 was evaluated in the supernatants of *in vitro* cultured splenocytes with an ELISA assay using the mouse IFN-γ, TNF-α, IL-2, IL-4, and IL-6 ELISA Set II kits (BD Pharmingen, San Jose, CA, USA and eBioscience, San Diego, CA, USA) according to the manufacturer’s instructions.

### Bronchoalveolar lavage (BAL) and leukocyte counts

The animals (6 per group)were euthanized by CO_2_ inhalation, and the trachea was surgically exposed and cannulated at two weeks after the last challenge. BAL fluid was collected from the mice by washing the lungs with 1 mL of PBS. The BAL fluid samples were centrifuged at 500 - *g* for 8 min at 4°C to obtain the supernatants. The total number of leukocytes (diluted with Turk’s 2% acetic acid solution) was counted using a Neubauer hemocytometer. Differential counts were performed in the cytospins stained by the May-Grunwald-Giemsa method. The counts are reported as the total number of cells per mL of BAL fluid.

### Lung permeability

Mice were sedated with 4% isoflurane in oxygen (9 per group). After we confirmed that mice were sedated, they were injected intravenously with 0.2 mL of 2% Evans Blue dye (Sigma) and were sacrificed one hour later. The lungs were collected and placed in 2 mL of formamide and incubated at 37°C overnight (VETEC, Duque de Caxias, RJ, Brazil) to extract the Evans Blue dye from the tissue. The absorbance was measured at λ = 620 nm (Molecular Devices Spectra Max 190). The Evans Blue dye concentration was calculated from a standard curve and is expressed as mg of Evans Blue dye per lung tissue.

### Quantification of BAL cytokines

The cytokine concentrations in the BAL fluid were measured using commercially available ELISA kits for TNF-α, IFN-γ, IL-4, and IL-6 (BD Pharmingen, San Jose, CA, USA) according to the manufacturer’s protocol.

### Histopathology

For the histopathology analysis, anesthetized mice (8 per group) were exsanguinated through the vena cava, and the lungs were inflated by injecting 1.0 mL of 10% buffered formalin through the same catheter that was used to perform the BAL. The lungs were then removed, fixed in 10% buffered formaldehyde, and paraffin embedded. Lung sections (5-μm thick) were stained with hematoxylin-eosin. The analysis of the tissue sections was performed using an Olympus BX41 light microscope at a magnification of 100X. The qualitative analysis of the tissue sections and of the captured images was carried out using a computer-assisted image analyzer (Image-Pro Plus Version 4.1 for Windows; Media Cybernetics, LP, Silver Spring, MD, USA). One observer who was unaware of the experimental conditions examined all of the tissue sections in a random order.

### Statistical analysis

The results are expressed as the mean values and the corresponding standard deviation of the individual results.

The normal distribution of the values of each variable was assessed by the Anderson Darling A2 test (Analyze-it). The statistical analysis was performed using SPSS software (for Windows). Comparisons between and within groups were performed by an analysis of variance test (one-way ANOVA) and Student-Newman-Keuls test (significance level of 0.050).

## Results

### Hydrostatic pressure inactivates the avian influenza virus H3N8

**Fig 1:**
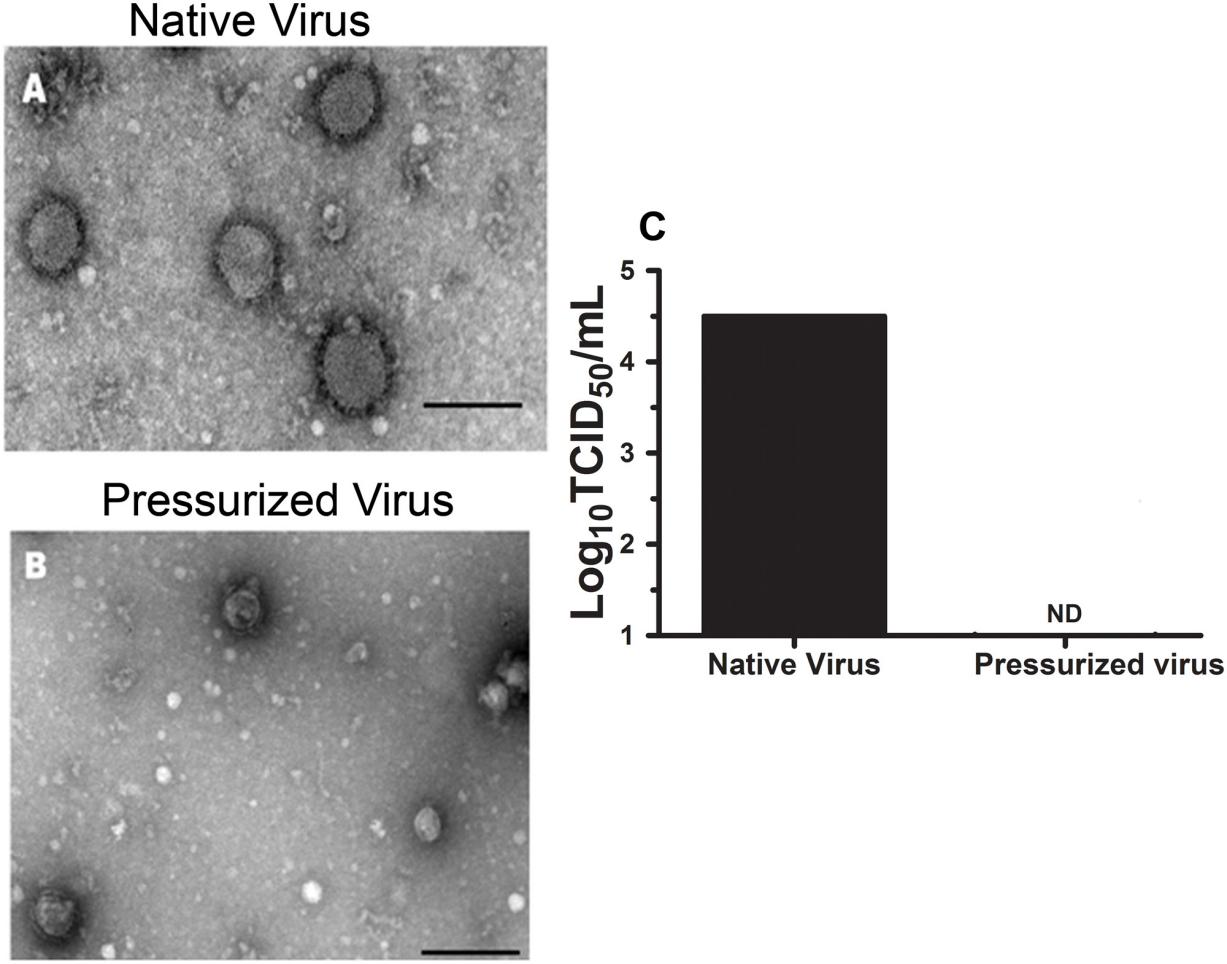
Inactivation of the avian influenza virus by hydrostatic pressure. Influenza virus was pressurized for 12 hours/282.7 MPa at 25°C. The H3N8 Influenza virus electron microscopy is shown. (A)shows the native virus, and (B) represents the pressurized virus sample. The arrow in B indicates the formation of a "pore" on the viral envelope. The selected micrographs are representative of all grids analyzed. Bars = 300 nm. In (C), evaluation of the pressurized virus infectivity is shown. In this assay, viruses were used at 100 μg/mL. The MDCK cell monolayers were infected with virus dilutions of 10^-1^ up to 10^-7^. The plates were incubated at 37°C, 5% CO_2_ for 48 hours. TCID_50_ values were calculated using the Reed-Muench method. ND = not detected by the method used.

Influenza A virus subtype H3N8, pressurized at 282.7 MPa for 12 hours, showed no infectivity as detected by a TCID_50_ assay (Fig 1). We next tested for potential residual infectivity with serial passages in embryonated chicken eggs and cultured MCDK cells. For each blind passage, no infectivity was detected by the hemagglutination or TCID_50_ assays (Table 1).

**Table 1:** Analysis of residual virus infectivity after pressure-induced inactivation^a^.

Sample	TCID_50_(log_10_)	Cytopathic effect in MDCK cells	Viral growth in embryonated eggs
**Saline**	ND^b^	-	-
**Native virus**	104.5	+	+
**Pressurized virus**	ND^b^	-	-

^a^ The residual infectivity of the pressurized virus samples (282.7 MPa for 12 h at 25°C) was assayed for three sequential serial passages in embryonated hens eggs and in MDCK cell monolayers. For each blind passage, the samples revealed the absence of infectivity by a hemagglutination assay or a TCID_50_.

^b^ No residual infectivity was detected in MDCK cells.

We could not associate the presence of the pressure-inactivated virus with any sign of disease (ruffled fur, lethargy, weight loss, and huddling) or mortality in the vaccinated animals. Infection with the native virus was able to kill 5-week-old mice and was associated with all of the disease signs investigated here. However, 9-week-old adult mice were not killed by the native virus despite showing all of the disease symptoms. Thus, effective pressure inactivation conditions were established as 282.7 MPa for 12 h at 25°C.

Fig 1 shows the transmission electron micrographs of influenza A H3N8 virus particles after incubation at atmospheric pressure or at 282.7 MPa (for 12 h, 25°C). The viruses treated with the hydrostatic pressure had a smaller size compared with the native viruses, and their shells were not as regular as the native shells.

To confirm viral inactivation we evaluated lung and nasal lavage samples from vaccinated animals. Infectious titers were not detected in the lungs of the analyzed animals, when the evaluation was performed according to TCID_50_. Nasal lavage was also evaluated by qRT-PCR, and the results showed the absence of infectious titers (Table 2).

**Table 2:** Virus titer measured in nasal wash and lung after vaccination^a^.

	Nasal wash(viral RNA levels (x 10^–12^ copies/mL))^b^		LungTCID_50_/mL(log_10_) ^b^	
	3 days	6 days	3 days	6 days
**Vaccinated**	ND^b^	ND^b^	ND^b^	ND^b^
**Saline**	ND^b^	ND^b^	ND^b^	ND^b^
**Challenged**	319.99 ± 10.07	410.02 ± 23.22	10^3^	10^1^

^a^ BALB/c mice were vaccinated with pressurized virus. Samples were collected 3 and 6 days after vaccination.

^b^ The results are expressed as the means of six animals.

### HP slightly affects envelope glycoproteins

**Fig 2:**
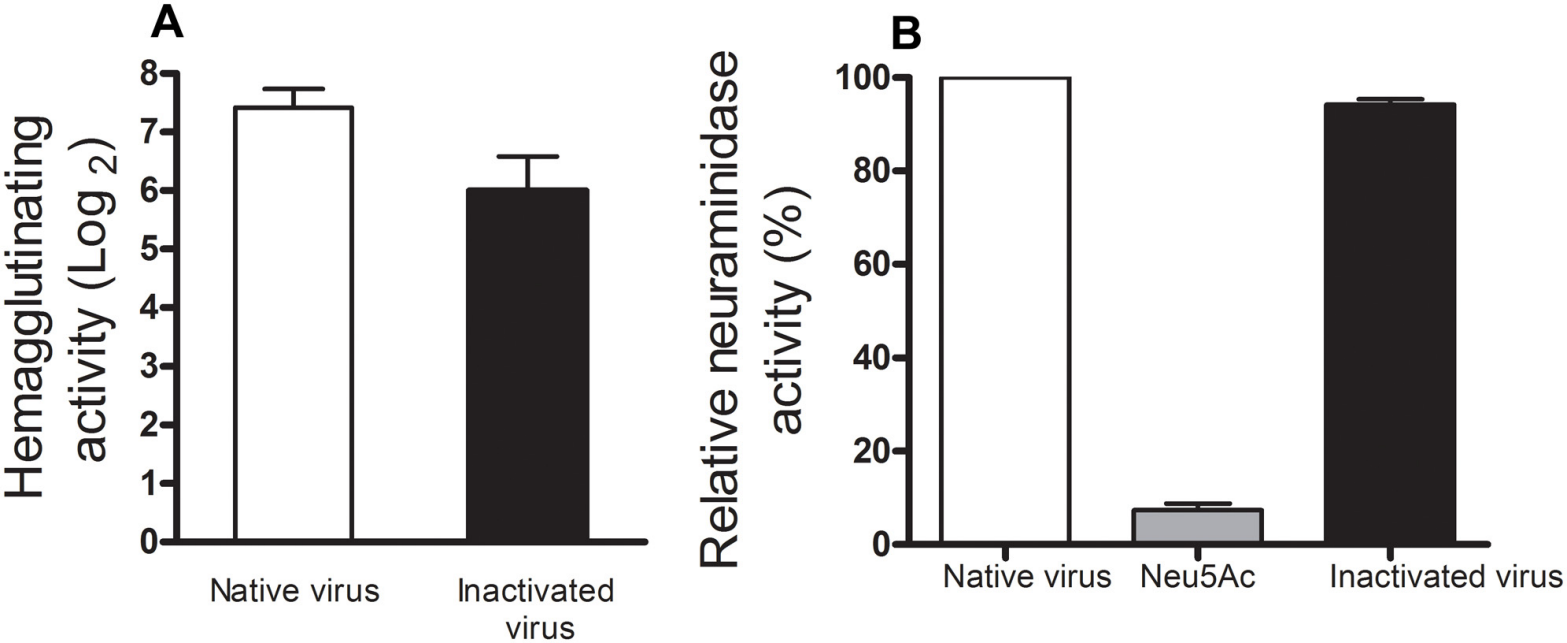
Activity of the surface glycoproteins in the pressure-inactivated influenza virus. (A) The hemagglutinating activity of H3N8 was tested as a function of the incubation time under pressure (282.7 MPa) at room temperature. The virus was pressurized for 12 hours at pH 7.4. For the assay, 50 μL of virus and 50 μL of 0.01 M phosphate buffered saline (PBS) were added to the wells. The contents of the wells in column 1 were serially diluted (1:2) through column 12, resulting in dilutions ranging from 1 to 1/1024. Fifty microliters of a 1% human blood cell suspension was added to all of the wells. The hemagglutination units (HAU) were calculated by the reciprocal of the highest dilution where complete hemagglutination was observed. (B) The virus was pressurized for 12 hours (282.7 MPa) at pH 7.4. The virus particles were diluted in 32.5 mM MES, pH 6.5, 4.0 mM CaCl_2_ prior to the assay such that the final concentration of virus was 20 μg/mL. N-acetylneuraminic acid (Neu5Ac) was used as negative control. All of the reactions were carried out in triplicate. The mean values of these replicates were used in the analysis of the data.

To test the effects of HP on viral binding activity, we evaluated the capacity of influenza virus to bind to erythrocytes by performing a hemagglutination assay. Even after 12 h of pressurization, the viruses were capable of binding to the cells, with a 2-log reduction in the titer (Fig 2A).

We also investigated neuraminidase activity. The NA activity was evaluated by testing the cleavage of the fluorometric substrate 4-MUNANA. We found that this pressure range slightly affected NA activity under the conditions tested (Fig 2B).

### Vaccinated mice are protected against infection

**Fig 3:**
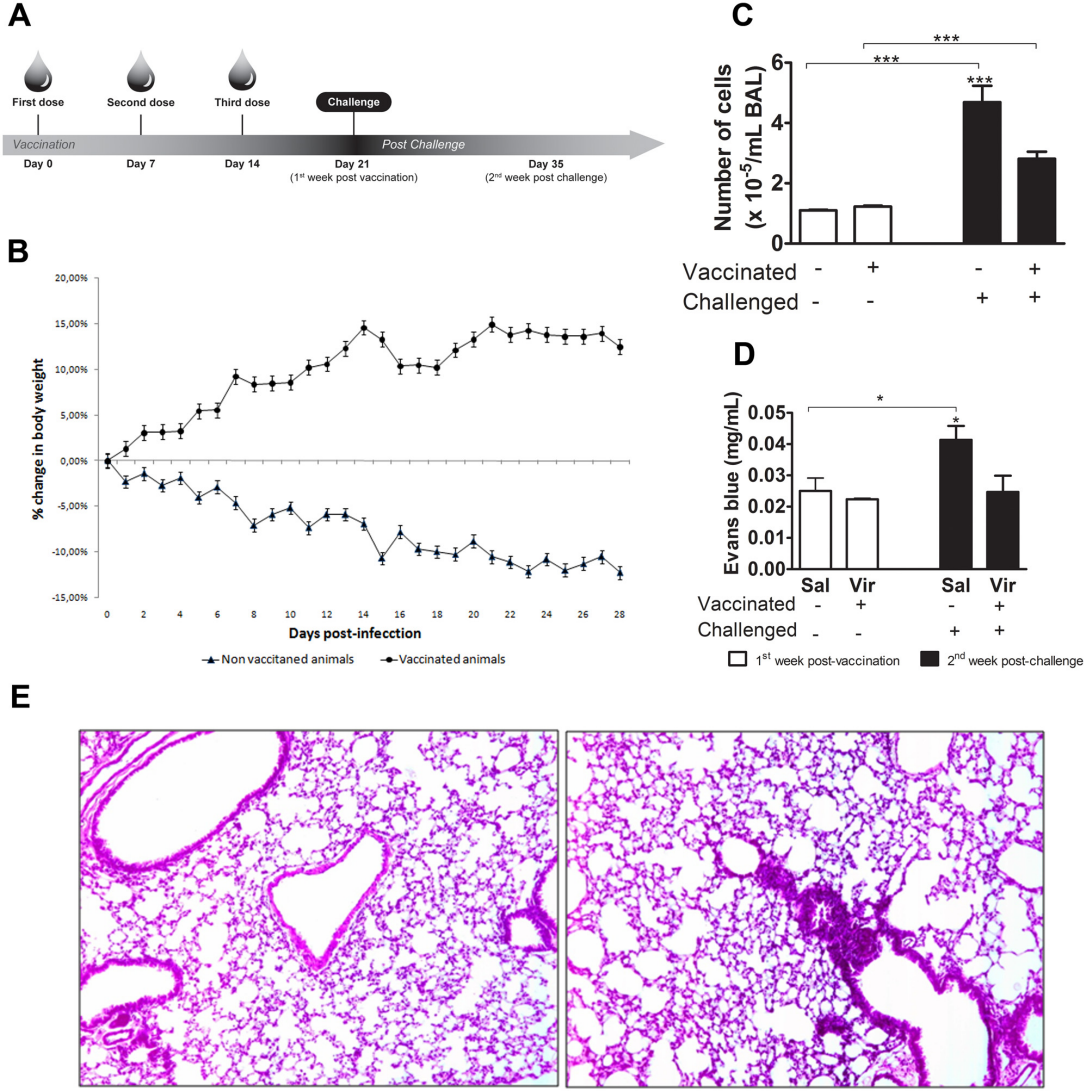
Vaccinated mice are protected against challenge. (A) Study design: BALB/c mice were vaccinated intranasally with the pressurized H3N8 at the indicated time points, followed by an intranasal challenge with the native virus. (B) The vaccinated and further challenged animals were monitored daily for body weight changes. The results are expressed as the mean of 10 animals. The mice were monitored for 28 days. (C) Total cell counts in the bronchoalveolar lavage (BAL) fluid are shown. At days 21 and 35 of the study, BAL was performed on the animals (n = 6) to analyze cellularity. The bars represent the averages and respective standard deviations of the individual results. (D) The measurement of Evans Blue dye in the lungs is shown. The animals (n = 9) were anesthetized and injected with an endovenous solution and 2% Evans Blue. After 1 hour, the animals were euthanized and perfused through the right atrium with physiological saline. Next, the lung was removed and placed in 5 mL of formamide for 18 h at 37°C. The supernatant was then collected and the absorbance of the Evans Blue dye was measured. The result was evaluated in mg/mL Evans Blue by making a standard curve. The bars represent the means and respective standard deviations of the individual results. (E) Histological sections of the lung parenchyma of the mice vaccinated and further challenged are shown. I: vaccinated challenged BALB/c, II: non-vaccinated challenged mice. The lungs were collected 7 days after challenge. The blocks were cut on a microtome to a thickness of 5 μm, and the slides were stained with hematoxylin and eosin for the morphological analysis. The slides were photographed using an Olympus BX41 microscope coupled to a photographic system at 100X magnification. Sal = Saline, Vir = Pressure-inactivated virus, PV = Post vaccination. P.I. = Post-infection with native virus. The asterisk (*) marks a significant difference (* p <0.05, ** p <0.01, *** p <0.0001 and a Student-Newman-Keuls post-test).

Using a mouse model, we investigated the immunogenic capacity and protective efficacy of the pressure-inactivated virus against infection. Fig 3A presents the time line of the experiments. To this end, we observed the mortality rates and clinical signs (ruffled fur, lethargy, weight loss, and huddling) in the mice. None of the vaccinated animals showed any weight loss or other clinical signs during the vaccination period (21 days). However, all of the non-vaccinated animals showed all of the clinical signs evaluated, but no death was observed. Challenging the vaccinated animals was not associated with weight loss during the observational period (28 days). However, challenging the non-vaccinated animals was associated with significant weight loss (p<0.05, day 20 post-challenge) (Fig 3B).

The cellular inflammatory response to influenza virus was assessed in the bronchoalveolar lavage (BAL) fluid of the vaccinated animals and the challenged vaccinated mice. The BAL was performed one week post-vaccination and two weeks post-challenge, as described in the Material and Methods section. Viable counts were performed to determine the total cell numbers. One week after the end of the vaccination period, we observed no difference in BAL fluid cell counts. After challenge, we observed a significant increase (p<0.001) in the BAL fluid total cell counts in the non-vaccinated mice compared with the vaccinated mice (Fig 3C).

Inflammation leads to changes in vascular integrity and permeability. To evaluate the alterations in lung vascular permeability we used the Evans Blue dye assay. Fig 3D shows that no alterations in the Evans Blue concentration were detected between the vaccinated and non-vaccinated mice. However, after challenge, the Evans Blue content in the lungs was significantly (p<0.05) increased in the non-vaccinated mice (Fig 3D).

The degree of the lung inflammation was also evaluated in hematoxylin and eosin-stained sections two weeks after challenge. The histological analysis showed increases in lung inflammation as revealed by the presence of perivascular inflammatory infiltrates in lung tissue sections from the non-vaccinated animals compared with the vaccinated animals (Fig 3E).

Lung homogenates were evaluated for TCID_50_ titers to determine whether vaccination with pressurized virus induced a protective immune response following challenge. At days 3 and 6 post-challenge, the vaccinated mice showed no detectable infectivity, whereas animals that were not vaccinated but challenged presented infectious titers of 10^3^ and 10^1^ TCID_50_, respectively (Table 3).

**Table 3:** Virus titer measured in lung homogenates after challenge^a^.

	TCID_50_/mL(log_10_)^b^	
	3 days Post-challenge	6 days Post-challenge
**Saline**	ND^c^	ND^c^
**Non-vaccinated/challenged**	10^3^	10^1^
**Vaccinated/challenged**	ND^c^	ND^c^

^a^ BALB/c mice were vaccinated with pressurized virus and challenged intranasally with native virus.

^b^ The results are expressed as the means of six animals.

^c^ No infectivity was detected in MDCK cells.

### Influenza vaccine is associated with increased antibody levels

**Fig 4:**
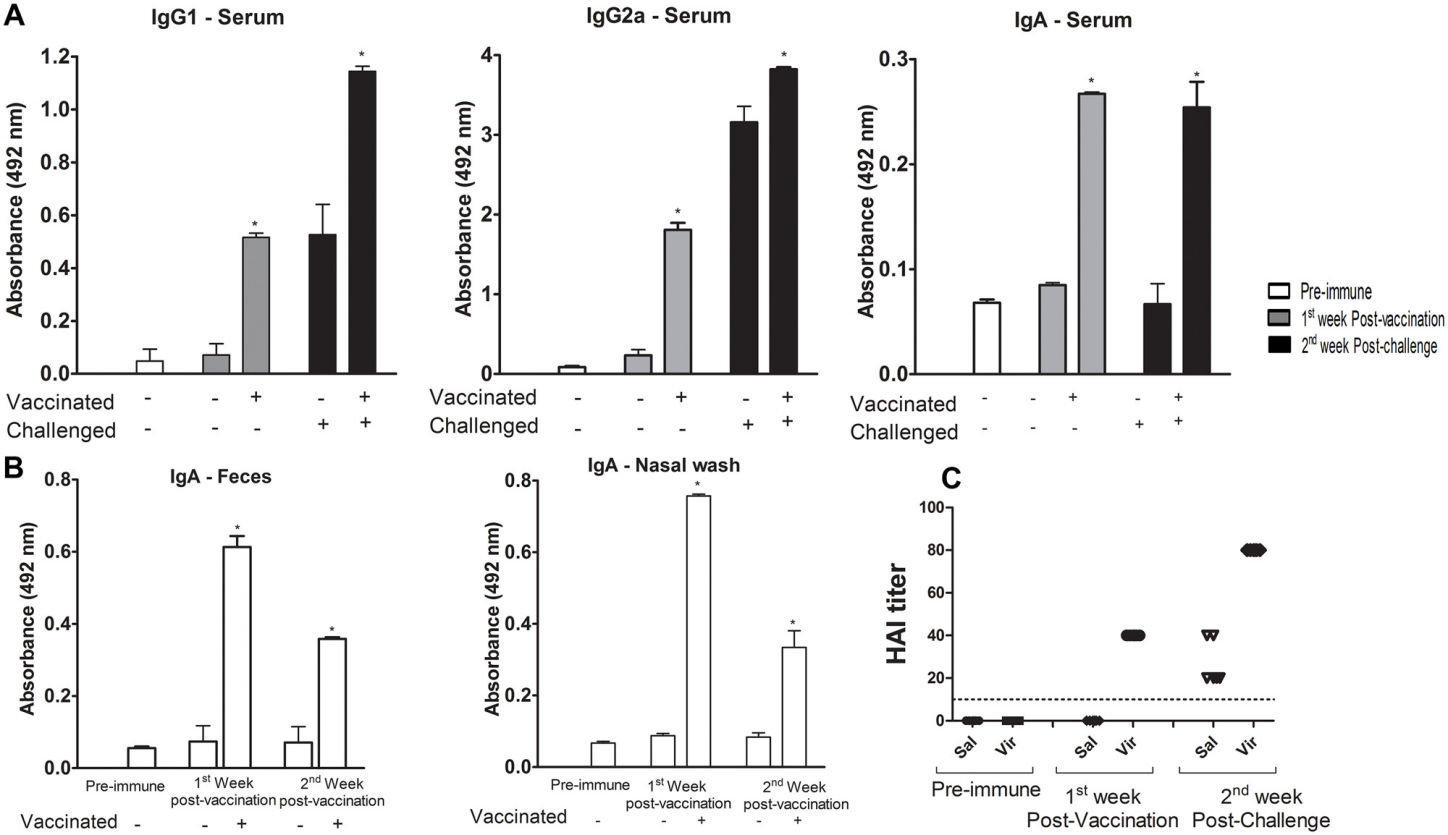
Induction of humoral immune responses and neutralizing antibodies by pressurized H3N8 after vaccination and challenge. (A) The influenza-specific IgG1, IgG2a, and IgA levels in serum at days zero, 21, and 35 according to the study design are shown. (B) Influenza-specific IgA in the nasal wash and feces at days zero and 21, according to the study design, are shown. After the third vaccination, followed one week later by the viral challenge, the antibodies were measured by ELISA using 2 μg of the antigen per well and goat anti-mouse IgG1, IgG2a, or IgA horseradish peroxidase-conjugated antibodies. The results are expressed as the mean of the absorbance values (492 nm) of the 1/1,000 diluted serum of each animal. (C) The response of the H3N8 neutralizing antibodies in sera is shown. The serum of the vaccinated and further challenged mice was assessed by a hemagglutination inhibition assay. The titer of the antibodies is referred to as the reciprocal of the highest serum dilution that resulted in the complete inhibition of the cytopathic effect. The dotted line represents the detection limit of the assay at a dilution of 10^10^. The symbols represent the result for each individual animal. Sal = Saline, Vir = Pressure-inactivated virus, P.I. = Post-infection with native virus (challenge). The asterisk (*) marks a significant difference (* p <0.05, ** p <0.01, p <0.0001 and a Student-Newman-Keuls post-test).

To investigate the capacity of pressure-inactivated virus to induce humoral immunity, we used an ELISA to measure the IgA and IgG subclasses of influenza-specific antibody responses. Higher levels of all of the antibodies were observed after the vaccination and challenge (Fig 4A), and similar results were obtained at all tested intervals (one, two, three and four weeks) between the last immunization and the challenge. The results for IgG1 were similar, and the levels of IgG2a were increased slightly with longer intervals (S1 Fig). The third dose of the vaccine, given at day 21, significantly boosted the IgG2a and IgG1 antibody titers in serum. IgA was the least robustly increased in serum but remained elevated even 12 weeks after vaccination (data not shown). The antibody response in serum followed the distribution pattern IgG2a > IgG1 > IgA.

IgA levels were also measured in nasal lavage and feces one week post-vaccination. We observed an increased level of IgA in the vaccinated mice. At 12 weeks after vaccination, the levels of IgA were still higher in the vaccinated animals than in the non-vaccinated animals (Fig 4B).

The ability of serum antibodies to neutralize the native virus was also tested in different intervals. After vaccination and challenge, all animals presented high seroprotective (≥1:40) HAI titers (Fig 4C and S2 Fig).

### Pressure-inactivated virus induces high IFN-γ secretion in the vaccinated mice and in the vaccinated and challenged mice

To further investigate the ability of the pressure-inactivated virus vaccine to stimulate the immune system, we evaluated the production of cytokines in splenocyte supernatants and BAL fluid.

In mice, the most common Th1 cytokines involved in the immune response are IFN-γ, IL-12, and IL-2, whereas IL-4, IL-5, IL-6, and IL-10 are associated with a Th2 response. A similar Th response pattern is observed in humans [40]. Thus, we evaluated cytokine production by analyzing the BAL fluid and the supernatants from splenocytes after *in vitro* H3N8 influenza virus stimulation.

**Fig 5:**
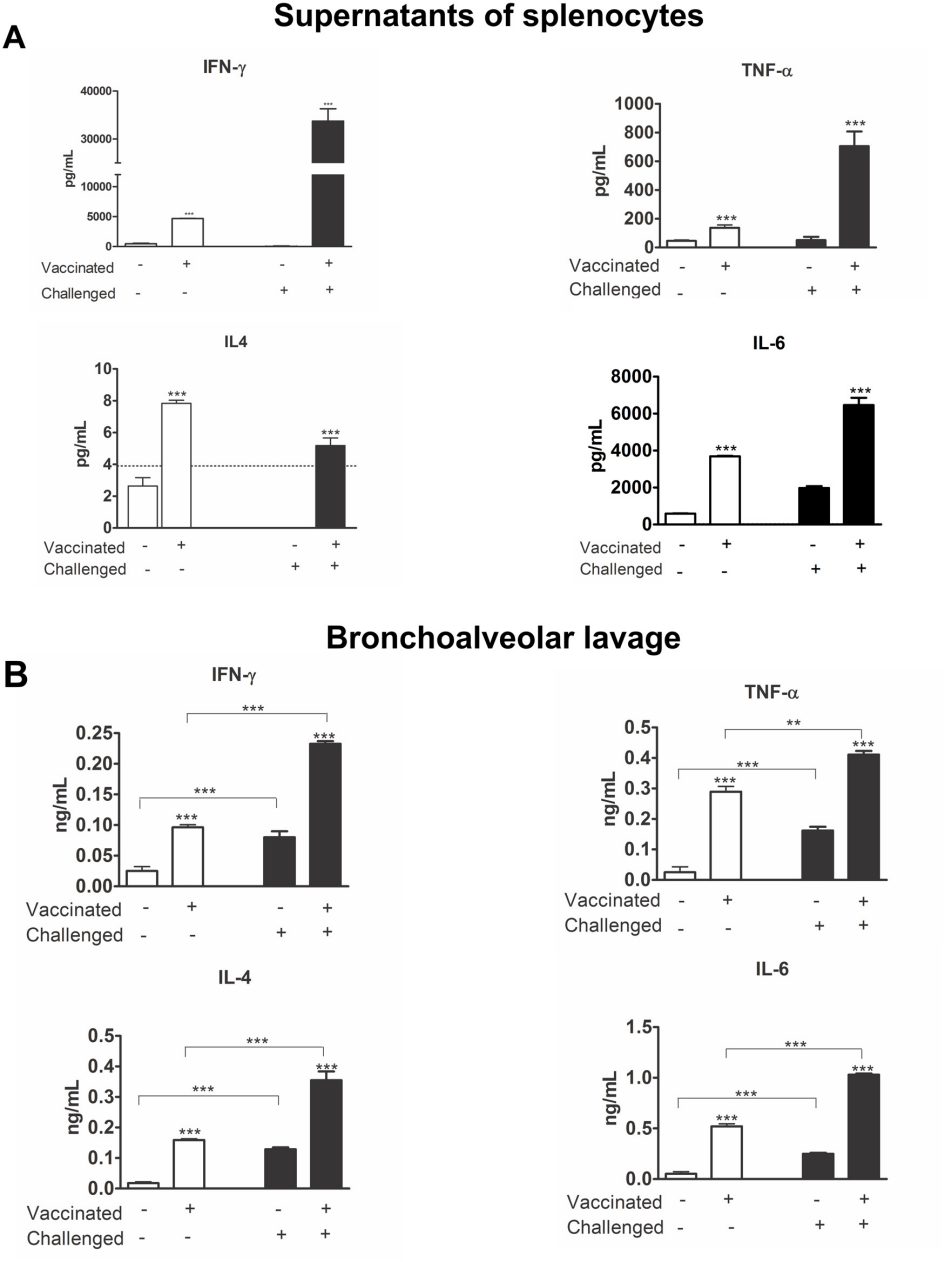
Induction of cytokine secretion in splenocyte supernatants and bronchoalveolar lavage fluid after vaccination and challenge. (Panel A) The concentration of the secreted cytokines in the supernatant of the splenocyte cultures *in vitro*, collected on days 21 and 35, according to the study design are shown. (Panel B) Cytokine levels were measured in BAL fluid collected on days 21 and 35. The bars represent the mean values and corresponding standard deviations of the individual results. All of the analyses were performed individually. Sal = Saline, Vir = Pressure-inactivated virus, P.V. = Post vaccination, P.I. = Post-infection with the native virus. The asterisk (*) marks a significant difference (* p <0.05, ** p <0.01, p <0.0001 and a Student-Newman-Keuls post-test).

One week post-vaccination, we observed an increase in all of the measured cytokines, primarily IFN-γ and IL-6 (p<0.001). Two weeks after challenge the IL-2, IFN-γ, TNF-α, and IL-6 levels were higher than the levels observed post-vaccination. We also measured IFN-γ, TNF-α, and IL-4 at 12 weeks after challenge, and only IFN-γ remained higher than the controls (data not shown) (Fig 5A). These data indicate that the cytokine response in the vaccinated animals and the challenged vaccinated animals was directed towards a Th1 response.

The evaluation of BAL fluid revealed that the post-vaccination production of cytokines was higher than in the controls (p<0.001). In the vaccinated-challenged animals, we observed the same pattern of increased cytokine production as was observed after the vaccination. However, IL-6 was especially high after challenge (Fig 5B).

## Discussion

In this study, we have shown that the intranasal vaccination of mice with an avian influenza virus inactivated by HP induced effective protection against infection.

Importantly, we demonstrate that the method used for virus inactivation in the production of a whole avian influenza virus vaccine induces not only the production of neutralizing antibodies in sera but also the production of IgA in nasal mucosa and in feces. Furthermore, we observed a marked IFN-γ response after vaccination.

We found that the pressurized virus particles were smaller than the native virus particles (Fig 1). These structural changes would be the cause of inactivation. Inactivation was assessed using a TCID_50_ assay and qRT-PCR (Tables 1 and 2). Previous studies have shown that human and animal viruses can be inactivated by HP [41,42]. Additionally, other authors have also shown changes in particle shape, membrane integrity, or capsid proteins [24,31]. To our knowledge this is the first study to investigate the humoral and cellular immunogenic response in vaccinated and challenged vaccinated animals with a virus inactivated by hydrostatic pressure.

We evaluated the effect of HP on HA and NA activity (Fig 2). The pressurization led to a drop of 2 logs_2_ in the hemagglutination test. We believe that this result is due to changes in the binding site of HA with N-acetylneuraminic acid. In 2003, Gomes *et al*. showed that HP led to structural changes in the membrane G protein from VSV. It was also demonstrated that the pressurized particles were capable of fusing with the cell membrane but unable to cause an infection [43]. More recently, our group showed similar results with the human H3N2 influenza virus [44]. These previous studies indicate that the inactivation by pressure preserves, even partially, the viral structure because the virus is able to bind to the target cell but is unable to enter the replication cycle [45].

In general, the pressure acts in the secondary structure, tertiary and quaternary structures of proteins, which are maintained by hydrophobic interactions, electrostatic and hydrogen bonds [46]. However, the literature shows that quaternary structures are the most affected [47,48]. Based on our data, we believe that the inactivation by HP changes the structure and activity of both hemagglutinin and neuraminidase, but preserving the generation of neutralizing response when the pressurized particles are used as a vaccine (Fig 4C and S2 Fig). More studies are being performed to determine the extension of these changes and whether the pressure-inactivated virus generates cross-protection.

We observed that the challenged non-vaccinated animals showed weight loss and had other clinical signs (Fig 3B), while the challenged vaccinated animals maintained their weight within the expected growth curve [49]. Furthermore, we found that challenged vaccinated animals showed no detectable viral titers by TCID_50_ assay (Table 3). These results suggest that the vaccine used in this study is effective at controlling the viral infection.

An influenza infection causes lung damage. This is associated with the infiltration of inflammatory cells and the increased leakage of proteins into the lung. In this study, we also analyzed lung damage by evaluating the cytokines and cellularity in BAL fluid and lung vascular permeability. We observed that the lungs of the challenged vaccinated animals showed a lower amount of blue dye compared with the challenged non-vaccinated animals, suggesting an effective action of the vaccine in controlling inflammation (Fig 3D). Moreover, these data are consistent with the results obtained by Gehlen *et al*. in mice, rats, and rabbits [50].

We also evaluated the vaccine-induced protection of the lung by histological analysis. The challenged non-vaccinated animals had lung inflammation, whereas the lung tissue of the challenged vaccinated animals had a normal appearance (Fig 3E). The lung inflammation in the challenged non-vaccinated animals was consistent with an influenza virus infection [51,52] even though the damage observed was milder than that observed using a mouse-adapted influenza virus or H5N1 [51,53].

The method most frequently used to evaluate the immunogenicity of a vaccine is the determination of antibody levels before and after vaccination [54]. Usually studies of immune response of vaccines start using BALB/c mice as model [55]. A vaccine response that resembles immune response to natural infection is considered the "gold standard" for protection [56].

We observed that the vaccination increased IgG2a levels and, to a lesser extent, IgG1 levels (Fig 4A). However, other authors have also found that lower levels of IgG2a, compared with those of IgG1, protect challenged animals [57]. Additionally, it is more desirable for the IgG2a subtype to be higher after a vaccination because it is linked to cross-protection [55]. Until now the hallmark for the efficacy of an influenza vaccine has been the induction of adequate levels of serum-neutralizing antibodies [56,58].

The influenza virus is spread in large quantities by excretion in the feces of birds [59]. Mucosal IgA secretion is the first line of pathogen defense. Thus, an increase in the IgA-specific viruses in the feces is an important observation (Fig 4B), as a positive correlation between the increase in the level of the mucosal IgA and the protection against a challenge has been reported [60,61]. We also observed an increase in the serum IgA in all of the vaccinated animals, regardless of challenge (Fig 3A). Serum IgA may act as a second line of defense [62], inducing phagocytosis by neutrophils [63]. Based on the work of Lu *et al*., who showed that the avian and human influenza viruses can infect mice and induce neutralizing antibodies detectable by the hemagglutination inhibition assay, we performed the test to verify that the antibodies found in the sera had a neutralizing capacity [64]. Our data show that all of the vaccinated animals had high protection titers (Fig 4C and S2 Fig) [65,66].

Studies of vaccines usually do not consider the production of cytokines [67]. However, it is known that native virus vaccines and whole virus vaccines induce similar responses, indicating that the structural integrity of the viral particles is important for the immune response [68]. We observed a mixed Th1/Th2 profile in the splenocyte supernatants. All of the vaccinated animals showed an increase in the secretion of all of the measured cytokines (Fig 5A). However, the high secretion of IFN-γ drives the immune response to a Th1 response. This pattern was still noted at 12 weeks after the infection (data not shown) and was also observed by Geeraedts *et al*. in 2008 [68]. The memory response against influenza is not completely understood. It is known that not only are memory B cells essential for generating antigen-specific antibodies but that memory T cells also play an essential role against reinfection with influenza virus [69].

Our IL-6, TNF-α, and IFN-γ results are in agreement with the literature, which states that whole virus vaccines induce high levels of these cytokines (reviewed by Reeth, 2000) [70]. IFN-γ is effective in inducing IgG2a and IgA in serum and protection against challenge [71], which corroborates our results. The increased concentrations of IL-6 and IL-10 could explain the presence of IgA at our dosages because these two cytokines are involved in the immunoglobulin class switch to IgA [72].

The analysis of BAL fluid is considered a sensitive method for the diagnosis of pulmonary inflammatory diseases [73]. The cytokine analysis in BAL fluid showed that in all of the vaccinated animals, an increase in all cytokines, particularly IL-6, took place (Fig 5B). Zosky *et al*. showed increased IL-6 and TNF-α in the BAL fluid of the challenged mice [74]. The increase of these cytokines in all of the vaccinated animals agrees with the literature showing an increase in the cytokines in BAL fluid of mice and humans after an influenza infection [75].

Our data show that the pressure-inactivated virus was able to generate neutralizing antibodies in serum, mucosal IgA, and strong IFN-γ response. Furthermore, the challenged animals showed no symptoms of disease and had better immune responses than the control animals.

Hydrostatic pressure is a well-established technique used in the food industry to eliminate bacteria and viruses from processed foods, such as canned products, milk, oysters, ham, and juice [76,77]. These technological developments increased the feasibility of commercial application in pathogens inactivation area [78]. Then, the establishment of an HP vaccine would not require technological innovations.

Besides, intranasal vaccination is non-invasive and prevents viral disease by blocking the site of entry, and this route also has the advantage of preventing horizontal transmission [79].

Although whole inactivated virus reactogenicity commonly raises concerns, a study in humans showed that there were no significant differences in the side effects between the split vaccine and the WIV groups. This study also suggested that despite the incidence of adverse effects in young children, the benefits of low doses may outweigh the risks [80].

Another advantage of the intranasal method is that the vaccine is easily administered and does not require skilled personnel. Thus, immunization campaigns adopting intranasal vaccines may be easily conducted in remote areas or in those areas with very limited resources, representing additional justification for developing a pressurized intranasal vaccine against influenza viruses.

Once all the viral components are present in the vaccine, the response observed in our results may be also influenced by the innate response [81,82]. Further investigation is necessary to elucidate the specific roles of the innate immune system and the adaptive immune system, or what pathways are triggered by vaccination with the pressure-inactivated virus.

## Supporting Information

S1 Fig Influenza-specific IgG1 and IgG2a levels at one, two, three and four weeks between the last vaccination and challenge. After the third vaccination, antibodies were measured with one, two, three and four weeks resting time between the last vaccination and the challenge. The control animals were inoculated with saline and challenged. The antibodies present in serum were measured by ELISA using 2 μg of the antigen per well and goat anti-mouse IgG1 or IgG2a horseradish peroxidase-conjugated antibodies. The results are expressed as the mean of the absorbance values (492 nm) of serum diluted 1/1,000 from each animal.
(TIF)

S2 Fig Induction of neutralizing antibodies by the pressurized H3N8 vaccine. The data show neutralizing antibodies in sera after four weeks resting time between the last vaccination and challenge, assessed by a hemagglutination inhibition assay. The titer of the antibodies is referred to as the reciprocal of the highest serum dilution that resulted in the complete inhibition of the cytopathic effect. The dotted line represents the detection limit of the assay at a dilution of 10^10^. The symbols represent the result for each individual animal. Sal = Saline, Vir = Pressure-inactivated virus, P.I. = Post-infection with native virus (challenge).
(TIF)
