# Supplementary figures and images for: Intranasal Immunization with Pressure Inactivated Avian Influenza Elicits Cellular and Humoral Responses in Mice
Source: PLoS One. 2015 Jun 9;10(6):e0128785. doi: 10.1371/journal.pone.0128785 (PMC4461174; doi:10.1371/journal.pone.0128785)

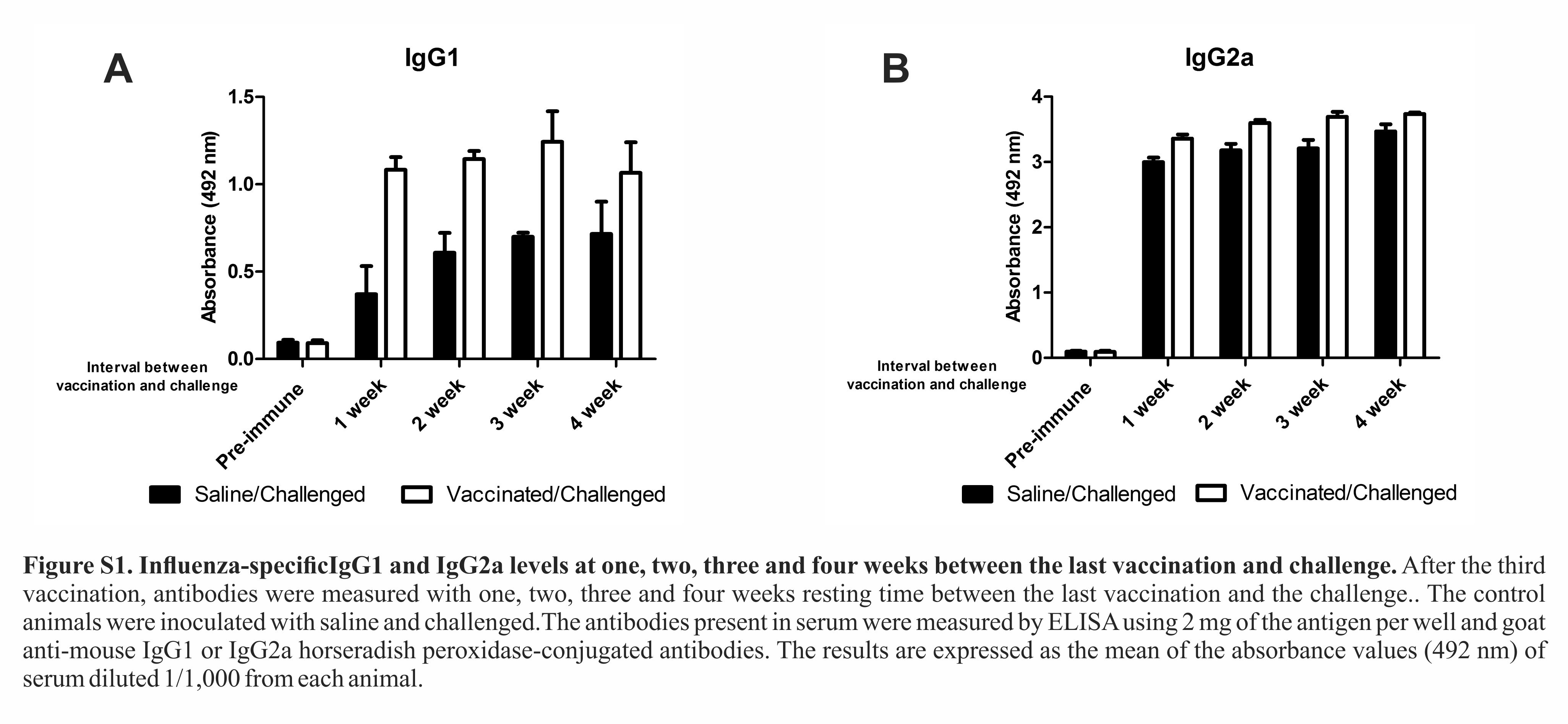

Supplement: S1 Fig — After the third vaccination, antibodies were measured with one, two, three and four weeks resting time between the last vaccination and the challenge. The control animals were inoculated with saline and challenged. The antibodies present in serum were measured by ELISA using 2 μg of the antigen per well and goat anti-mouse IgG1 or IgG2a horseradish peroxidase-conjugated antibodies. The results are expressed as the mean of the absorbance values (492 nm) of serum diluted 1/1,000 from each animal. (TIF) [file pone.0128785.s001.tif]

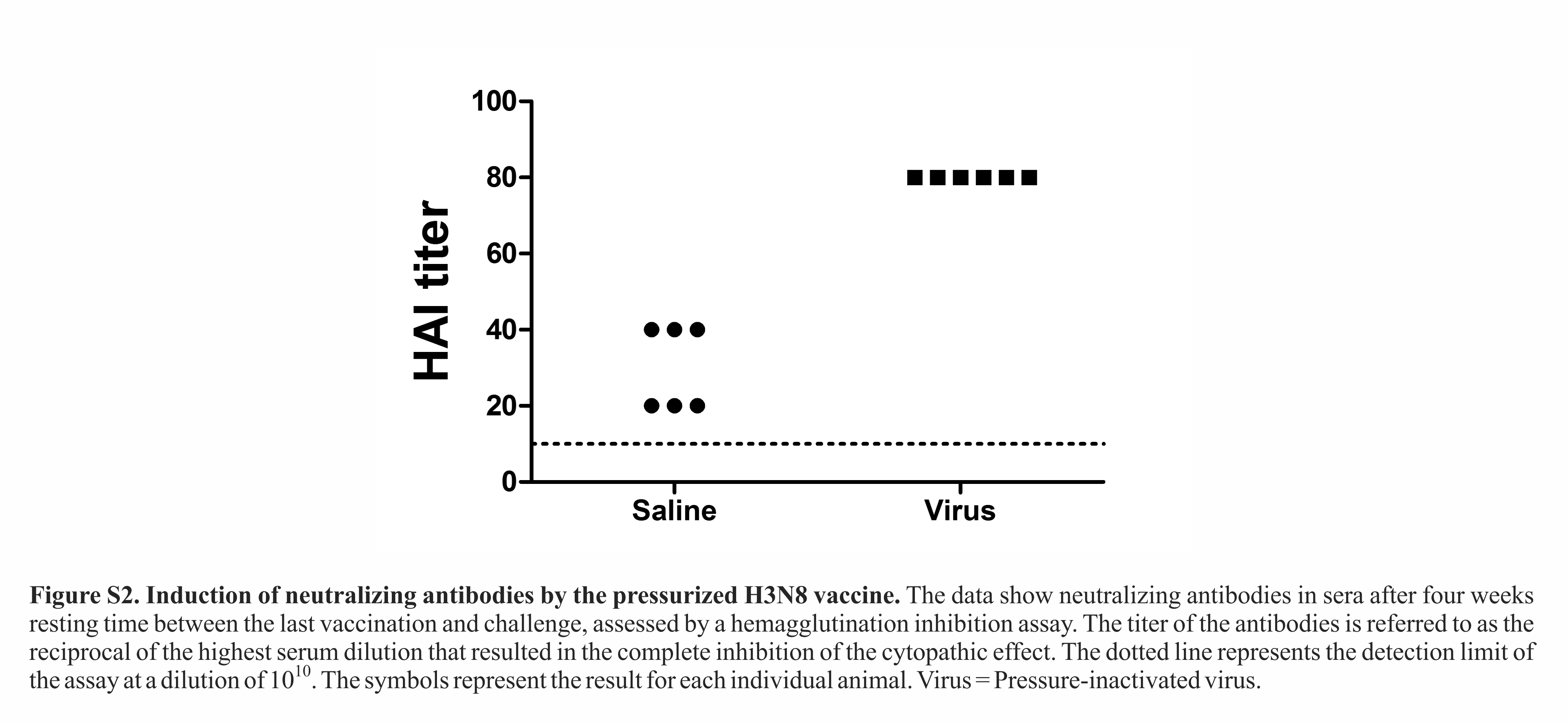

Supplement: S2 Fig — The data show neutralizing antibodies in sera after four weeks resting time between the last vaccination and challenge, assessed by a hemagglutination inhibition assay. The titer of the antibodies is referred to as the reciprocal of the highest serum dilution that resulted in the complete inhibition of the cytopathic effect. The dotted line represents the detection limit of the assay at a dilution of 1010. The symbols represent the result for each individual animal. Sal = Saline, Vir = Pressure-inactivated virus, P.I. = Post-infection with native virus (challenge). (TIF) [file pone.0128785.s002.tif]
